# Transitions and Instabilities in Imperfect Ion-Selective Membranes

**DOI:** 10.3390/ijms21186526

**Published:** 2020-09-07

**Authors:** Jarrod Schiffbauer, Evgeny Demekhin, Georgy Ganchenko

**Affiliations:** 1 Department of Physical and Environmental Sciences, Colorado Mesa University, Grand Junction, CO 81501, USA; jschiffbauer@coloradomesa.edu; 2 Department of Mathematics and Computer Science, Financial University, 350051 Krasnodar, Russia; 3 Laboratory of Micro- and Nanoscale Electro- and Hydrodynamics, Financial University, 350051 Krasnodar, Russia; ganchenko.ru@gmail.com; 4 Laboratory of General Aeromechanics, Institute of Mechanics, Moscow State University, 119192 Moscow, Russia

**Keywords:** ion-selective surface, electrokinetic instability, electroconvection, Darcy-Brinkman approach, 47.61.Fg, 47.57.jd, 82.45.-h, 82.39.Wj

## Abstract

Numerical investigation of the underlimiting, limiting, and overlimiting current modes and their transitions in imperfect ion-selective membranes with fluid flow through permitted through the membrane is presented. The system is treated as a three layer composite system of electrolyte-porous membrane-electrolyte where the Nernst–Planck–Poisson–Stokes system of equations is used in the electrolyte, and the Darcy–Brinkman approach is employed in the nanoporous membrane. In order to resolve thin Debye and Darcy layers, quasi-spectral methods are applied using Chebyshev polynomials for their accumulation of zeros and, hence, best resolution in the layers. The boundary between underlimiting and overlimiting current regimes is subject of linear stability analysis, where the transition to overlimiting current is assumed due to the electrokinetic instability of the one-dimensional quiescent state. However, the well-developed overlimiting current is inherently a problem of nonlinear stability and is subject of the direct numerical simulation of the full system of equations. Both high and low fixed charge density membranes (low- and high concentration electrolyte solutions), acting respectively as (nearly) perfect or imperfect membranes, are considered. The perfect membrane is adequately described by a one-layer model while the imperfect membrane has a more sophisticated response. In particular, the direct transition from underlimiting to overlimiting currents, bypassing the limiting currents, is found to be possible for imperfect membranes (high-concentration electrolyte). The transition to the overlimiting currents for the low-concentration electrolyte solutions is monotonic, while for the high-concentration solutions it is oscillatory. Despite the fact that velocities in the porous membrane are much smaller than in the electrolyte region, it is further demonstrated that they can dramatically influence the nature and transition to the overlimiting regimes. A map of the bifurcations, transitions, and regimes is constructed in coordinates of the fixed membrane charge and the Darcy number.

## 1. Introduction

Problems of electrokinetics and micro- and nanofluidics have recently attracted a great deal of attention due to rapid developments in micro-, nano-, and biotechnology. Among the numerous modern micro- and nanofluidic applications of electrokinetics are micropumps, micromixers, micro total analysis systems (μTAs), desalination, fuel cells, etc. (see [1]). Microscale processes are highly dependent on confining the flow surface. Ion-selective surfaces such as electrodes, ion exchange membranes, systems of micro- and nanochannels, etc. are very promising from the viewpoint of applications. One of the most promising applications of ion-selective surfaces is medical diagnostics [2,3,4].

The study of the space charge in the electric double-ion layer (EDL) in an electrolyte solution near an ion-selective surface under a potential drop is a fundamental problem in modern physics, first addressed by Helmholtz. The interest in the problem, in particular, is connected with a novel type of electrohydrodynamic instability—electrokinetic instability which determines transition to the overlimiting currents (OLC). Despite the extreme practical importance of the problem, its investigation in a full formulation has not yet been completed. One reason is that the membrane system is a complex, composite system consisting of electrolyte layers separated by a porous medium with internal structure, fixed surface charge and other functional groups which can even couple to the solution chemistry [5].

An important characteristic of the electric membranes, voltage-current (VC) curve, has three distinguishable regions [6,7]. (i) For a small drop of potential, the VC curve obeys a linear Ohmic relationship where the electric current is proportional to the voltage. This is the so called underlimiting regime theoretically described by Levich [6]; the solution is one-dimensional (1D) and only electrodiffusion is involved in this solution. To get a quite accurate analytical solution, only the electrolyte region need be considered. (ii) With voltage increasing, the limiting current regime occurs with the saturation region of the VC-curve. This regime is not described by the Levich solution and it was explained and studied numerically by Rubinstein and Shtilman [8]; this solution is also one-dimensional and hydrodynamics is not involved in it. For extreme values of the potential drop, this solution can be described by simple asymptotic analytical expansions [9,10]. (iii) With further increasing of the voltage, the saturation region ends and the electric current again linearly depends on the voltage; this regime is called overlimiting one. The electrokinetic instability as the main mechanism of transition to the overlimiting regimes was discovered in [11,12], where analytical solution of the linear stability problem was studied. Direct numerical simulation of the electrokinetic instability and the overlimiting regimes was fulfilled in [13,14,15,16,17,18,19]. In these works, in particular, details of time evolution, space distribution of the ion concentrations, the electric potential, the charge concentration, and the velocity field were investigated. Moreover, the VC characteristics were built up theoretically. In the overlimiting regime, the hydrodynamics become a key mechanism of ion transport and must be included in the solution, which can now be either two-dimensional (2D) at relatively small voltage, or three-dimensional (3D) at larger voltages.

All the authors mentioned above considered only elecrtolyte region and neglected the influence of the porous membrane, treating it as a perfect charge-selective surface that completely prohibits one kind of ion from penetrating through it. For perfect membranes, there is only one mechanism of instability that is caused by the nonequilibrium electro-osmotic slip of the second kind, the galvano-osmotic instability, and is connected with the nonuniformity of the electric current along the membrane; see [11,12]. From the viewpoint of perfect membranes, the underlimiting regimes are always stable (see Zholkovskiy et al. [20]), and a loss of stability and transition to the overlimiting currents occurs at the limiting current given sufficient overvoltage and the VC-characteristic has all three mentioned segments: the underlimiting, limiting, and overlimiting currents.

The properties of an imperfect membrane can be very different from its perfect analog. A mathematical formulation for the imperfect ion-selective membrane was first put forth in [21] and exploited in the works in [22,23,24,25,26]. In all the mentioned works only the electrostatic properties of an electric membrane were taken into account and the fluid inside the porous membrane is considered immobile. However, critically, both kinds of ions can penetrate the interface and diffusive transport becomes important; hence, the membrane system is imperfect. It was found that for the imperfect membrane surface, the loss of stability can occur for the underlimiting currents, bypassing the limiting currents [22]. Hence, the VC-curve can consist of two segments instead of three: the underlimiting and the overlimiting currents.

To date, there has not been a single study that takes into account the effect of nonzero hydrodynamic velocities within a porous membrane on the electrohydrodynamic response without. The reason for this is velocities in the membrane are considerably smaller than the velocities in the adjacent fluid; hence, the hydrodynamics inside the porous membrane seems reasonably negligible (see monograph by Zabolotsky and Nikonenko [27]). Moreover, such accounting greatly complicates the formulation and solution of the problem.

We point out that in addition to ion-selective membrane systems, composite systems consisting of a porous medium and an adjacent fluid layer are used in various engineering applications: drying processes, solid-matrix heat exchangers, electronics cooling, thermal insulation, heat pipes, nuclear reactors, and porous journal bearings. Mathematical models of such systems are well-developed and applied to numerous flows. An important for us result of these investigations is that even small velocities in the porous medium can have a significant effect on the loss of stability of the whole system, see for example [28]. Hence, it stands to reason to expect the same kind of influence in the membrane composite system; this fact inspired the present work.

For correct analysis of any composite flow, imposition of appropriate boundary conditions at the interface liquid-porous medium is crucial. For that reason, many investigators have proposed different types of interfacial conditions between the porous medium and the adjacent fluid layer, as summarized and compared in the work of Alazmi and Vafai [29]. There are two commonly recognized approaches to composite systems. The first one to match the momentum equations in the fluid-porous medium inter-region is to make use of an empirical expression proposed by Beavers and Joseph [30]. This expression relates the rate of strain in the fluid phase to the difference between the tangential velocities in the fluid and the porous media adjacent to the inter region. These boundary conditions require determination of an empirical parameter.

In the second approach, Ochoa-Tapia and Whitaker [31,32] presented a mathematical model for the jump of the shear stresses that involves Brinkman’s correction [33] to Darcy’s law in the porous layer. The method of volume averaging is used to derive a stress jump boundary condition. In this case, the differential equations that govern the momentum transport in both the fluid and porous layers are rendered in the same order so that it is possible to match the rates of strain in both regions. The stress-jump condition has an inherent problem; it involves an unknown coefficient β, which needs to be fitted either experimentally or numerically; it is shown to depend on the membrane texture: the porosity, the Darcy number and the pore characteristic size. Comparison of these two approaches (see, for example [34]) shows that they gives qualitatively rather similar results. The objective of the present work is to apply the Ochoa-Tapia and Whitaker [31,32] approach to the ion-selective membrane problem, following Kuznetsov [35] and take β as an independent parameter. This formulation and details on the numerical procedure are presented in the next section, followed by a discussion of the main results and conclusions.

## 2. Formulation

### 2.1. Dimensional Equations

**Figure 1:**
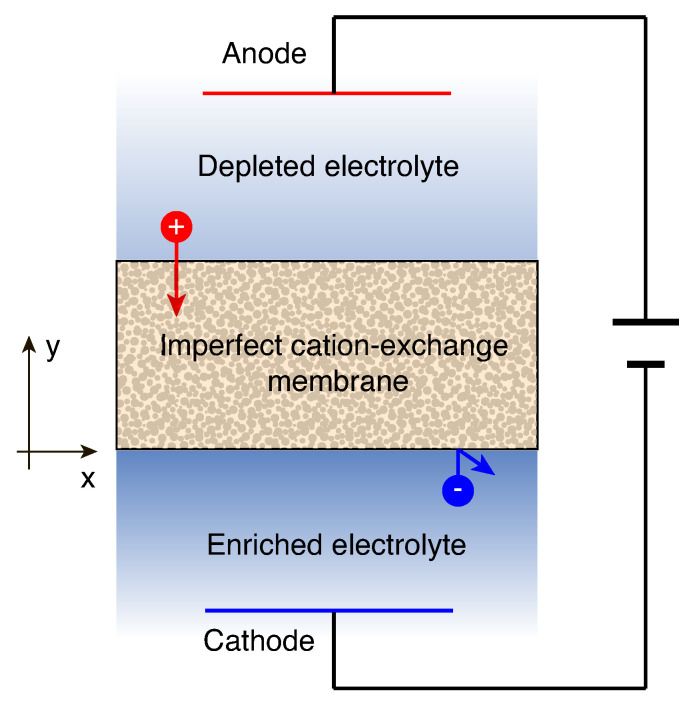
(Color online) Schematic of the membrane system, taken as a computation domain.

Let us consider a three layer system occupying the space $-\tilde{h}<\tilde{y}<2\tilde{h}$, in which there is fluid in the regions $-\tilde{h}<\tilde{y}<0$ and $\tilde{h}<\tilde{y}<2\tilde{h}$ overlying a porous cation exchange membrane region $0<\tilde{y}<\tilde{h}$ saturated with the same fluid (see Figure 1). Notations with tilde are used for the dimensional variables, as opposed to their dimensionless counterparts without tilde. $\left(\tilde{x},\tilde{y}\right)$ are the Cartesian coordinates, where $\tilde{x}$ is directed along the membrane interface and $\tilde{y}$ is normal to it. The fluid is assumed to be a symmetric (valence or charge number, $z^{+}=-z^{-}=1$), binary electrolyte with an equal diffusivity of cations and anions $\tilde{D}$, dynamic viscosity $\tilde{\mu }$, and electric permittivity $\tilde{\varepsilon }$.

The ion transport equations, the Poisson equation, and the Stokes equation for the creeping flow in the fluid are as follows,

(1) $$\begin{array}{c} \frac{\partial {\tilde{c}}^{\pm }}{\partial \tilde{t}}+\tilde{U}\frac{\partial {\tilde{c}}^{\pm }}{\partial \tilde{x}}+\tilde{V}\frac{\partial {\tilde{c}}^{\pm }}{\partial \tilde{y}}=\pm \frac{\tilde{D}\tilde{F}}{\tilde{R}\tilde{T}}\left[\frac{\partial }{\partial \tilde{x}}\left({\tilde{c}}^{\pm }\frac{\partial \tilde{\Phi }}{\partial \tilde{x}}\right)+\frac{\partial }{\partial \tilde{y}}\left({\tilde{c}}^{\pm }\frac{\partial \tilde{\Phi }}{\partial \tilde{y}}\right)\right]+\tilde{D}\left[\frac{\partial^{2}{\tilde{c}}^{\pm }}{\partial {\tilde{x}}^{2}}+\frac{\partial^{2}{\tilde{c}}^{\pm }}{\partial {\tilde{y}}^{2}}\right], \end{array}$$

(2) $$\tilde{\varepsilon }\left(\frac{\partial^{2}\tilde{\Phi }}{\partial {\tilde{x}}^{2}}+\frac{\partial^{2}\tilde{\Phi }}{\partial {\tilde{y}}^{2}}\right)=\tilde{F}\left({\tilde{c}}^{-}-{\tilde{c}}^{+}\right),$$

(3) $$\frac{\partial \tilde{\Pi }}{\partial \tilde{x}}-\tilde{\mu }\left(\frac{\partial^{2}\tilde{U}}{\partial {\tilde{x}}^{2}}+\frac{\partial^{2}\tilde{U}}{\partial {\tilde{y}}^{2}}\right)=\tilde{\varepsilon }\frac{\partial \tilde{\Phi }}{\partial \tilde{x}}\left(\frac{\partial^{2}\tilde{\Phi }}{\partial {\tilde{x}}^{2}}\frac{\partial^{2}\tilde{\Phi }}{\partial {\tilde{y}}^{2}}\right),$$

(4) $$\frac{\partial \tilde{\Pi }}{\partial \tilde{y}}-\tilde{\mu }\left(\frac{\partial^{2}\tilde{V}}{\partial {\tilde{x}}^{2}}+\frac{\partial^{2}\tilde{V}}{\partial {\tilde{y}}^{2}}\right)=\tilde{\varepsilon }\frac{\partial \tilde{\Phi }}{\partial \tilde{y}}\left(\frac{\partial^{2}\tilde{\Phi }}{\partial {\tilde{x}}^{2}}\frac{\partial^{2}\tilde{\Phi }}{\partial {\tilde{y}}^{2}}\right),$$

(5) $$\frac{\partial \tilde{U}}{\partial \tilde{x}}+\frac{\partial \tilde{V}}{\partial \tilde{y}}=0,$$

where ${\tilde{c}}^{\pm }$, $\left(\tilde{U},\tilde{V}\right)$, $\tilde{\Pi }$ and $\tilde{\Phi }$ are molar concentrations of ions, velocity, pressure and electric potential respectively; $\tilde{F}$ is the Faraday number, $\tilde{R}$ is the universal gas constant, and $\tilde{T}$ is the absolute temperature.

Let us consider the porous membrane flanked into the electrolyte fluid. For the sake of simplicity, the porosity of the membrane is taken equal to unity, the viscosity, permittivity and diffusivity of the porous medium are assumed the same as their analogues in the fluid layer. Then the ion transport and the Poisson equations in the porous layer $0<\tilde{y}<\tilde{h}$ can be written as,

(6) $$\begin{array}{c} \frac{\partial {\tilde{c}}^{\pm }}{\partial \tilde{t}}+\tilde{U}\frac{\partial {\tilde{c}}^{\pm }}{\partial \tilde{x}}+\tilde{V}\frac{\partial {\tilde{c}}^{\pm }}{\partial \tilde{y}}=\pm \frac{\tilde{D}\tilde{F}}{\tilde{R}\tilde{T}}\left[\frac{\partial }{\partial \tilde{x}}\left({\tilde{c}}^{\pm }\frac{\partial \tilde{\Phi }}{\partial \tilde{x}}\right)+\frac{\partial }{\partial \tilde{y}}\left({\tilde{c}}^{\pm }\frac{\partial \tilde{\Phi }}{\partial \tilde{y}}\right)\right]+\tilde{D}\left[\frac{\partial^{2}{\tilde{c}}^{\pm }}{\partial {\tilde{x}}^{2}}+\frac{\partial^{2}{\tilde{c}}^{\pm }}{\partial {\tilde{y}}^{2}}\right], \end{array}$$

(7) $$\tilde{\varepsilon }\left(\frac{\partial^{2}\tilde{\Phi }}{\partial {\tilde{x}}^{2}}+\frac{\partial^{2}\tilde{\Phi }}{\partial {\tilde{y}}^{2}}\right)=\tilde{F}\left({\tilde{c}}^{-}-{\tilde{c}}^{+}\right)+\tilde{F}\tilde{N},$$

where ${\tilde{c}}^{\pm }$, $\left(\tilde{U},\tilde{V}\right)$, $\tilde{\Pi }$ and $\tilde{\Phi }$ are molar concentrations of ions, velocity, pressure, and electric potential respectively, and $\tilde{N}$ is the fixed charge density. As the membrane is defined to be a cation exchange membrane, $\tilde{N}$ is positive.

The governing equations for the hydrodynamics are those of the Darcy–Brinkman model in the Stokes approximation, corrected by the Coulomb forces in the right hand side of the momentum equations,

(8) $$\frac{\partial \tilde{\Pi }}{\partial \tilde{x}}+\frac{\tilde{\mu }}{\tilde{\kappa }}\tilde{U}-\tilde{\mu }\left(\frac{\partial^{2}\tilde{U}}{\partial {\tilde{x}}^{2}}+\frac{\partial^{2}\tilde{U}}{\partial {\tilde{y}}^{2}}\right)=\tilde{\varepsilon }\frac{\partial \tilde{\Phi }}{\partial \tilde{x}}\left(\frac{\partial^{2}\tilde{\Phi }}{\partial {\tilde{x}}^{2}}+\frac{\partial^{2}\tilde{\Phi }}{\partial {\tilde{y}}^{2}}\right),$$

(9) $$\frac{\partial \tilde{\Pi }}{\partial \tilde{y}}+\frac{\tilde{\mu }}{\tilde{\kappa }}\tilde{V}-\tilde{\mu }\left(\frac{\partial^{2}\tilde{V}}{\partial {\tilde{x}}^{2}}+\frac{\partial^{2}\tilde{V}}{\partial {\tilde{y}}^{2}}\right)=\tilde{\varepsilon }\frac{\partial \tilde{\Phi }}{\partial \tilde{y}}\left(\frac{\partial^{2}\tilde{\Phi }}{\partial {\tilde{x}}^{2}}+\frac{\partial^{2}\tilde{\Phi }}{\partial {\tilde{y}}^{2}}\right),$$

(10) $$\frac{\partial \tilde{U}}{\partial \tilde{x}}+\frac{\partial \tilde{V}}{\partial \tilde{y}}=0,$$

where $\tilde{\kappa }$ is a permeability of the porous material. For the sake of simplicity the porosity of the membrane is taken equal to 1.

This system must be complemented by the proper boundary conditions (BCs). With respect to the hydrodynamics boundary conditions, we assume the continuity of the velocity components and normal stresses,

(11) $$\tilde{U}{|}_{\tilde{y}=\tilde{h}+0}=\tilde{U}{|}_{\tilde{y}=\tilde{h}-0},\;\tilde{V}{|}_{\tilde{y}=\tilde{h}+0}=\tilde{V}{|}_{\tilde{y}=\tilde{h}-0},\;\left(-\tilde{\Pi }+2\tilde{\mu }\frac{\partial \tilde{V}}{\partial \tilde{y}}\right){|}_{\tilde{y}=\tilde{h}+0}=\left(-\tilde{\Pi }+2\tilde{\mu }\frac{\partial \tilde{V}}{\partial \tilde{y}}\right){|}_{\tilde{y}=\tilde{h}-0},$$

(12) $$\tilde{U}{|}_{\tilde{y}=-0}=\tilde{U}{|}_{\tilde{y}=+0},\;\tilde{V}{|}_{\tilde{y}=-0}=\tilde{V}{|}_{\tilde{y}=+0},\;\left(-\tilde{\Pi }+2\tilde{\mu }\frac{\partial \tilde{V}}{\partial \tilde{y}}\right){|}_{\tilde{y}=-0}=\left(-\tilde{\Pi }+2\tilde{\mu }\frac{\partial \tilde{V}}{\partial \tilde{y}}\right){|}_{\tilde{y}=+0}$$

at the interfaces $\tilde{y}=\tilde{h}$ and $\tilde{y}=0$.

According to the Darcy–Brinkman model, which is proposed by Ochoa-Tapia and Whitaker [31,32], the tangential stresses have a jump, crossing $\tilde{y}=\tilde{h}$ and $\tilde{y}=0$,

(13) $$\tilde{\mu }\left(\frac{\partial \tilde{U}}{\partial \tilde{y}}+\frac{\partial \tilde{V}}{\partial \tilde{x}}\right){|}_{\tilde{y}=\tilde{h}+0}-\tilde{\mu }\left(\frac{\partial \tilde{U}}{\partial \tilde{y}}+\frac{\partial \tilde{V}}{\partial \tilde{x}}\right){|}_{\tilde{y}=\tilde{h}-0}=-\frac{\tilde{\mu }\beta }{\sqrt{\tilde{\kappa }}}\tilde{U}{|}_{\tilde{y}=\tilde{h}-0},$$

(14) $$\tilde{\mu }\left(\frac{\partial \tilde{U}}{\partial \tilde{y}}+\frac{\partial \tilde{V}}{\partial \tilde{x}}\right){|}_{\tilde{y}=-0}-\tilde{\mu }\left(\frac{\partial \tilde{U}}{\partial \tilde{y}}+\frac{\partial \tilde{V}}{\partial \tilde{x}}\right){|}_{\tilde{y}=+0}=-\frac{\tilde{\mu }\beta }{\sqrt{\tilde{\kappa }}}\tilde{U}{|}_{\tilde{y}=+0}.$$

Here β is an empirical constant which depends on the porosity, the Darcy number and the characteristic pore size. In the present work we adopt Kuznetsov’s approach [35] and assume β is an independent parameter, −1≤β≤1.

Let us consider the BCs for the electrostatics and the ion concentration. The electric potential, the concentration of cations and anions and their the fluxes, the electric field normal to the interfaces $\tilde{y}=0$ and $\tilde{y}=\tilde{h}$, are all continuous at the interfaces $\tilde{y}=0$ and $\tilde{y}=\tilde{h}$. Under our assumptions these conditions turn into the following,

(15) $$\tilde{\Phi }{|}_{\tilde{y}=\tilde{h}+0}=\tilde{\Phi }{|}_{\tilde{y}=\tilde{h}-0},\;\frac{\partial \tilde{\Phi }}{\partial \tilde{y}}{|}_{\tilde{y}=\tilde{h}+0}=\frac{\partial \tilde{\Phi }}{\partial \tilde{y}}{|}_{\tilde{y}=\tilde{h}-0},\;{\tilde{c}}^{\pm }{|}_{\tilde{y}=\tilde{h}+0}={\tilde{c}}^{\pm }{|}_{\tilde{y}=\tilde{h}-0},\;\frac{\partial {\tilde{c}}^{\pm }}{\partial \tilde{y}}{|}_{\tilde{y}=\tilde{h}+0}=\frac{\partial {\tilde{c}}^{\pm }}{\partial \tilde{y}}{|}_{\tilde{y}=\tilde{h}-0},$$

(16) $$\tilde{\Phi }{|}_{\tilde{y}=-0}=\tilde{\Phi }{|}_{\tilde{y}=+0},\;\frac{\partial \tilde{\Phi }}{\partial \tilde{y}}{|}_{\tilde{y}=-0}=\frac{\partial \tilde{\Phi }}{\partial \tilde{y}}{|}_{\tilde{y}=+0},\;{\tilde{c}}^{\pm }{|}_{\tilde{y}=-0}={\tilde{c}}^{\pm }{|}_{\tilde{y}=+0},\;\frac{\partial {\tilde{c}}^{\pm }}{\partial \tilde{y}}{|}_{\tilde{y}=-0}=\frac{\partial {\tilde{c}}^{\pm }}{\partial \tilde{y}}{|}_{\tilde{y}=+0}.$$

At the outer boundary of the diffusion layers, $\tilde{y}=-\tilde{h}$ and $\tilde{y}=2\tilde{h}$, the so called reservoir conditions [11] are applied:

(17) $$\tilde{y}=-\tilde{h}:\;\tilde{\Phi }=0,\;{\tilde{c}}^{\pm }={\tilde{c}}_{\infty },\;\tilde{V}=0,\;\frac{\partial \tilde{U}}{\partial \tilde{y}}+\frac{\partial \tilde{V}}{\partial \tilde{x}}=0,$$

(18) $$\tilde{y}=2\tilde{h}:\;\tilde{\Phi }=\Delta \tilde{V},\;{\tilde{c}}^{\pm }={\tilde{c}}_{\infty },\;\tilde{V}=0,\;\frac{\partial \tilde{U}}{\partial \tilde{y}}+\frac{\partial \tilde{V}}{\partial \tilde{x}}=0,$$

where $\Delta \tilde{V}$ is a fixed potential drop.

An important characteristic of the electrodialysis cell is the electric current density through the interface $\tilde{y}=\tilde{h}$, which is determined by the flux of cations and anions,

(19) $$\tilde{F}\left({\tilde{j}}^{+}-{\tilde{j}}^{+}\right)=\frac{{\tilde{F}}^{2}\tilde{D}}{\tilde{R}\tilde{T}}\left({\tilde{c}}^{+}+{\tilde{c}}^{-}\right)\frac{\partial \tilde{\Phi }}{\partial \tilde{y}}+\tilde{F}\tilde{D}\frac{\partial ({\tilde{c}}^{+}-{\tilde{c}}^{-})}{\partial y}+\tilde{F}\tilde{V}\left({\tilde{c}}^{+}-{\tilde{c}}^{-}\right).$$

Adding initial conditions for the cations and anions completes the system (1)–(19). These initial conditions arise from the following viewpoint: when there is no potential difference $\Delta \tilde{V}$, the distribution of ions is homogeneous and neutral,

(20) $$\tilde{t}=0:\;{\tilde{c}}^{\pm }={\tilde{c}}_{\infty }+“\mathrm{room disturbances}”.$$

The neutral conditions are corrupted by a small environmental random noise, so called “room disturbances”.

### 2.2. Dimensionless Equations

To render the Equations (1)–(20) dimensionless, the following characteristic quantities are used:
$\tilde{h}$:the porous membrane thickness;$\frac{{\tilde{h}}^{2}}{\tilde{D}}$:the characteristic time;$\frac{\tilde{D}}{\tilde{h}}$:the characteristic velocity;$\tilde{\mu }$:viscosity is taken as the characteristic dynamic value;$\frac{\tilde{\mu }\tilde{D}}{{\tilde{h}}^{2}}$:the characteristic stress;${\tilde{\Phi }}_{0}=\frac{\tilde{R}\tilde{T}}{\tilde{F}}$:the characteristic thermal voltage;${\tilde{c}}_{\infty }$:unperturbed ion concentration far from the diffusion layer;$\frac{\tilde{D}\tilde{F}{\tilde{c}}_{\infty }}{\tilde{h}}$:the characteristic electric current.

Here, $\tilde{F}$ is the Faraday constant, $\tilde{R}$ is the universal gas constant, and $\tilde{T}$ is the absolute temperature.

The problem is described by the following non-dimensional equations,

(21) $$\frac{\partial c^{\pm }}{\partial t}+U\cdot \nabla c^{\pm }=\pm \nabla \cdot \left(c^{\pm }\nabla \Phi \right)+\nabla^{2}c^{\pm },\;\nu^{2}\nabla^{2}\Phi =c^{-}-c^{+}+\gamma N,$$

(22) $$\nabla \Pi +\frac{\gamma }{Da^{2}}U-\nabla^{2}U=\kappa \nabla^{2}\Phi \cdot \nabla \Phi ,\;\nabla \cdot U=0.$$

Here γ=1 in the porous membrane, 0<y<1, and γ=0 in the fluid region, −1<y<0 and 1<y<2.

Upon introducing of the stream function Ψ, U=∂Ψ/∂y and V=−∂Ψ/∂x and excluding the pressure, the Stokes equations turn into the one biharmonic equation with the Darcy and Coulomb corrections,

(23) $$\nabla^{4}\Psi -\frac{\gamma }{Da^{2}}\nabla^{2}\Psi =\kappa \frac{\partial \Phi }{\partial y}\cdot \frac{\partial }{\partial x}\nabla^{2}\Phi -\kappa \frac{\partial \Phi }{\partial x}\cdot \frac{\partial }{\partial y}\nabla^{2}\Phi ,$$

with the BCs at the upper boundary of the electrodialysis system, y=2:

(24) $$y=2:\;c^{\pm }=1,\;\frac{\partial \Psi }{\partial x}=0,\;\frac{\partial^{2}\Psi }{\partial y^{2}}-\frac{\partial^{2}\Psi }{\partial x^{2}}=0,\;\Phi =\Delta V,$$

the BCs on the lower boundary, y=−1:

(25) $$y=-1:\;c^{\pm }=1,\;\frac{\partial \Psi }{\partial x}=0,\;\frac{\partial^{2}\Psi }{\partial y^{2}}-\frac{\partial^{2}\Psi }{\partial x^{2}}=0,\;\Phi =0,$$

(26) $$y=2:\;c^{\pm }=1,\;\frac{\partial \Psi }{\partial x}=0,\;\frac{\partial^{2}\Psi }{\partial y^{2}}-\frac{\partial^{2}\Psi }{\partial x^{2}}=0,\;\Phi =\Delta V,$$

the BCs at the electrolyte-membrane interface, y=1,

(27) $$\frac{\partial \Psi }{\partial x}{|}_{y=1+0}=\frac{\partial \Psi }{\partial x}{|}_{y=1-0},\;\frac{\partial \Psi }{\partial y}{|}_{y=1+0}=\frac{\partial \Psi }{\partial y}{|}_{y=1-0}$$

(28) $$\left(\frac{\partial^{2}\Psi }{\partial y^{2}}-\frac{\partial^{2}\Psi }{\partial x^{2}}\right){|}_{y=1+0}-\left(\frac{\partial^{2}\Psi }{\partial y^{2}}-\frac{\partial^{2}\Psi }{\partial x^{2}}\right){|}_{y=1-0}=-\frac{\beta }{Da}\frac{\partial \Psi }{\partial y}{|}_{y=1-0}$$

(29) $$\left(\frac{\partial^{3}\Psi }{\partial y^{3}}+3\frac{\partial^{3}\Psi }{\partial x^{2}\partial y}\right){|}_{y=1+0}-\left(\frac{\partial^{3}\Psi }{\partial y^{3}}+3\frac{\partial^{3}\Psi }{\partial x^{2}\partial y}-\frac{1}{Da^{2}}\frac{\partial \Psi }{\partial y}\right){|}_{y=1-0}=\kappa \frac{N}{\nu^{2}}\frac{\partial \Phi }{\partial x}{|}_{y=1-0}$$

(30) $$\Phi {|}_{y=1+0}=\Phi {|}_{y=1-0},\;\frac{\partial \Phi }{\partial y}{|}_{y=1+0}=\frac{\partial \Phi }{\partial y}{|}_{y=1-0},\;c^{\pm }{|}_{y=1+0}=c^{\pm }{|}_{y=1-0},\;\frac{\partial c^{\pm }}{\partial y}{|}_{y=1+0}=\frac{\partial c^{\pm }}{\partial y}{|}_{y=1-0}.$$

Similar with (27)–(30), BCs must be set at the boundary y=0.

The fluxes of cations and anions at the interface y=1 can be obtained from the following relations,

(31) $$y=1:\;j^{+}=c^{+}\frac{\partial \Phi }{\partial y}+\frac{\partial c^{+}}{\partial y}-c^{+}\frac{\partial \Psi }{\partial x},\;j^{-}=c^{-}\frac{\partial \Phi }{\partial y}-\frac{\partial c^{-}}{\partial y}+c^{-}\frac{\partial \Psi }{\partial x},$$

giving the density of the electric current as the difference between these two fluxes as follows,

(32) $$y=1:\;j^{+}-j^{-}=\left(c^{+}-c^{-}\right)\frac{\partial \Phi }{\partial y}+\frac{\partial }{\partial y}\left(c^{+}+c^{-}\right)-\left(c^{+}+c^{-}\right)\frac{\partial \Psi }{\partial x}.$$

Adding initial conditions for the cations and anions (20) in the dimensionless form completes the system,

(33) $$t=0:\;c^{\pm }=1+“\mathrm{room disturbances}”.$$

The problem is described by the following six dimensionless parameters:
$\Delta V=\frac{\Delta \tilde{V}}{{\tilde{\Phi }}_{0}}$:the potential drop;$\nu =\frac{{\tilde{\lambda }}_{D}}{\tilde{h}}$:the Debye number;$Da=\frac{\sqrt{\tilde{\kappa }}}{\tilde{h}}$:the Darcy number;$N=\frac{\tilde{N}}{{\tilde{c}}_{\infty }}$:the dimensionless fixed charge density;$\kappa =\frac{\tilde{\varepsilon }{\tilde{\Phi }}_{0}^{2}}{\tilde{\mu }\tilde{D}}$:the coupling coefficient between the hydrodynamics and electrodynamics;β:the Brinkman coefficient,
where ${\tilde{\lambda }}_{D}=\sqrt{\tilde{\varepsilon }{\tilde{\Phi }}_{0}/{\tilde{c}}_{\infty }\tilde{F}}$ is the Debye length, the coupling coefficient κ depends only on the physical properties of the electrolyte.

Just to provide perspective, typical values of dimensional and dimensionless parameters are given below. The typical bulk concentration varies within the window, ${\tilde{c}}_{\infty }=1-10^{3}$ mol/m^3^ and the typical potential drop is about $\Delta \tilde{V}=0-5V$. The diffusivity is taken $\tilde{D}=10^{-9}$ m^2^/s. κ is fixed for a given electrolyte but typically lies between 0.05 to 0.5. In this work it is taken, κ=0.1. The fixed charge concentration $\tilde{N}$ inside the membrane depends on the type of membrane used and is usually written in the membrane passport; for regular membranes used for desalination, this value is of the order of $\tilde{N}=$$10^{3}$ to $2\times 10^{3}$ mol/m^3^ (see Ref. [27].) The membrane thickness $\tilde{h}$ for regular desalination devices is on the order of 0.5 mm, however, for some other devices in use, the thickness can be much smaller, micrometers and even down to 10 nm, see Ref. [21]. The Debye length ${\tilde{\lambda }}_{D}$, depending on the concentration ${\tilde{c}}_{\infty }$, lies within the window 1 to 100 nm; accepted regular typical value is 10 nm. The permeability $\tilde{\kappa }$ varies within $\tilde{\kappa }=10^{-18}$ m^2^ to $\tilde{\kappa }=10^{-12}$ m^2^, hence, the porous characteristic size changes in the same window as the Debye length. It also means that the problem has two small parameters, the Debye number $\nu ={\tilde{\lambda }}_{D}/\tilde{h}$ and the Darcy number $Da=\sqrt{\tilde{\kappa }}/\tilde{h}$.

In this case, the dimensionless parameters change in the following windows: ΔV=0−500, $\nu =10^{-5}-10^{-3}$, $Da=0-5\times 10^{-2}$, N=0.1−20, and β varies from −1 to +1. Results of calculations depend only weakly on the Debye number (see [12,14,15,16]), and so it will be fixed in our calculations, $\nu =5\times 10^{-4}$. The Brinkman coefficient β, properly speaking, is not an independent parameter, and it depends on the texture of the membrane, namely, on the porosity, the Darcy number, and the characteristic pore size. It must be found either experimentally for a given texture of the membrane or using special numerical calculations. In order to avoid these painstaking procedures, we adopted Kuznetsov’s approach [35], where −1<β<1 was taken as an independent parameter. Fortunately, similar to the case in Ref. [35], we found that dependence on β is very weak and most calculations were made for β=0. In subsequent calculations, three dimensionless parameters are varied, namely, ΔV, *N* and Da, and we explain this weak dependence on β.

### 2.3. Numerical Method and Its Justification

**Figure 2:**
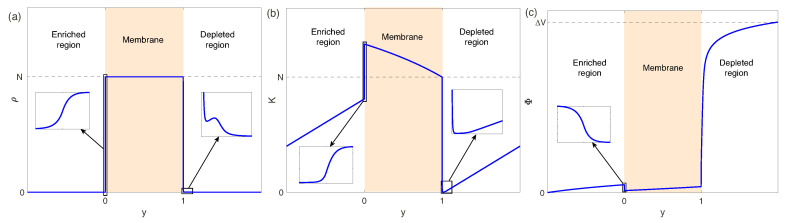
(Color online) Expected distributions of the charge density $\rho =c^{+}-c^{-}$ (**a**), the salt concentration $K=c^{+}+c^{-}$ (**b**) and the electric potential Φ (**c**) in the normal to the electrolyte-membrane surface.

For a wide parameter range the problem (21)–(33) can be solved only numerically. The main difficulties in the numerical solution arise in the direction normal to the electrolyte-membrane surface, the *y*-direction. In the electrolyte, near the electrolyte-membrane interface, a thin Debye layer, O(ν), is formed. This layer is connected with a small coefficient in the Poisson equation. Moreover, there ia also a small coefficient in the Brinkman-Darcy equation, the Darcy number. Thus another thin layer, O(Da), arises in the membrane, near the interface. For perfect membranes, with a large fixed charge *N*, an effect of the charge jump upon crossing the interface must also be taken into account. Small layer thickness leads to rapid changing of the unknowns and large spatial derivatives. The expected distributions of the unknowns are shown in Figure 2a: The charge density $\rho =c^{+}-c^{-}$ has a large jump when crossing the electrolyte-membrane interface, caused by large fixed charge *N* in the membrane. Moreover, for the depleted electrolyte region the singularity is strengthened by the space charge region with a fast variation of ρ. As it is seen from Figure 2b, the salt concentration $K=c^{+}+c^{-}$ jump in the EDLs is nearly vertical and is close to zero in the depleted region; these facts also must be taken into account during choosing of the numerical method and the grid of the discretization. Variation of the electric potential Φ, Figure 2c, is much stronger in the depleted region and it is becoming more pronounced for larger ΔV. The presented behavior gives a hint to select a proper numerical method and proper discretization.

The finite-difference methods show poor performance and efficiency for such thin boundary layers and such rapid jumps. Usually in such cases, mesh stretching near singularities causes stiffness of the system, unacceptably small time step, and even possible failure of numerical convergence. The best method to overcome these difficulties is to apply spectral and quasi-spectral methods for the space discretization (see Canuto et al. [36]), recommended for investigation of the laminar-turbulent transitions and even a well-developed turbulence for Navier–Stokes equations (see Spalart [37]). For the perfect electric membranes a quasi-spectral method was successfully applied in Ref. [13]. Chebyshev’s orthogonal polynomials with accumulation of zeros and, hence, the best resolution near the boundaries, are chosen as the Galerkin basis. The τ-modification of the spectral method is employed to satisfy the boundary conditions.

Three kinds of problems are considered in this work, (a) one-dimensional (1D) steady solution for the underlimiting and limiting currents, (b) transition to the overlimiting currents, and (c) well-developed overlimiting regimes. Finding the one-dimensional solution and investigating its linear stability (problems (a) and (b)) require only discretization in *y*-direction. However, the direct numerical simulation to solve problem (c) also needs discretization along *x*-direction, which is tangential to the electrolyte-membrane surface. It stands to reason to also apply a quasi-spectral method in the *x*-direction by choosing the Fourier series as basis functions.

## 3. Results

### 3.1. The Underlimiting and Limiting Currents

The one-dimensional (1D) steady-state, ∂/∂x=∂/∂t=0, solution of the system (21)–(33) describes both underlimiting and limiting current regimes. The tangential electric field, ∂Φ/∂x, for such solutions is absent and the hydrodynamic flow is not involved in the solution. Both regimes are the result of competition between electromigration. The solution does not depend on the coupling coefficient κ and the Darcy number Da; it is function of the potential drop ΔV, the fixed charge *N* and the Debye number ν.

The system (21)–(33) can be transformed into a system of ordinary differential equations. For the all three layers, Equations (21) can be integrated once. Taking into account boundary conditions (27)–(31), after long and tedious derivations this system was transformed into the following three equations,

(34) $$\nu^{2}\frac{d^{3}\Phi }{dy^{3}}-\left[\frac{\nu^{2}}{2}{\left(\frac{d\Phi }{dy}\right)}^{2}+\left(j^{+}+j^{-}\right)\left(y-y_{m}^{\left(k\right)}\right)-N^{\left(k\right)}\Phi \right]\frac{d\Phi }{dy}+j^{+}-j^{-}=0.$$

Here index k=1,2,3 refers to one of three layers, the fixed space charge in the electrolyte layers is absent, $N^{\left(1\right)}=N^{\left(3\right)}=0$, $N=N^{\left(2\right)}$ and $y_{m}^{\left(k\right)}$ are constants of integration. The boundary conditions (24) and (30) turn into the ones,

(35) $$y=-1:\;\Phi =0,\;\frac{d^{2}\Phi }{dy^{2}}=0,\;y=2:\;\Phi =\Delta V,\;\frac{d^{2}\Phi }{dy^{2}}=0.$$

At the interfaces y=0 and y=1 function Φ and its first derivatives dΦ/dy and $d^{2}\Phi /dy^{2}$ are taken continuous; it follows from the original conditions (30), which, in turn, are valid when permittivity of the membrane ${\tilde{\varepsilon }}_{m}$ and its diffusivity ${\tilde{D}}_{m}$ are equal to the permittivity $\tilde{\varepsilon }$ and diffusivity $\tilde{D}$ of the electrolyte, respectively.

Three different expansions in the Chebyshev series were exploited in the layers. Upon substitution of these series into Equation (34) and into boundary conditions of their continuity and boundary conditions (35), we obtained a system of nonlinear algebraic equations with respect to unknown coefficients of the expansion. Depending on the voltage ΔV from 20 to 150 terms of the expansion were taken, so that the largest order of the nonlinear system was about 500 equations.

Our system of equations, $f=(f_{1},f_{2},\ldots ,f_{n})$, can be considered as a system of parameterized, nonlinear algebraic equations,

(36) f(ν,ΔV,N,x)=0,

where $x=(x_{1},x_{2},\ldots ,x_{n})$ is a vector of unknowns and the Debye number ν, the voltage ΔV, and the fixed charge *N* are the parameters of the system. Newton’s method was used to solve Equation (36). When the solution x for some parameters ν,ΔV was found, this solution was extended to the solution with close parameters, etc. The previous solution was taken as the initial approximation for the search for the next one and eventually to find the whole family. When Φ(y) and its first derivatives were known, the ion concentrations $c^{\pm }$, the charge density $\rho =c^{+}-c^{-}$, the salt concentration $K=c^{+}+c^{-}$, and the ion fluxes $j^{\pm }$ were readily found.

**Figure 3:**
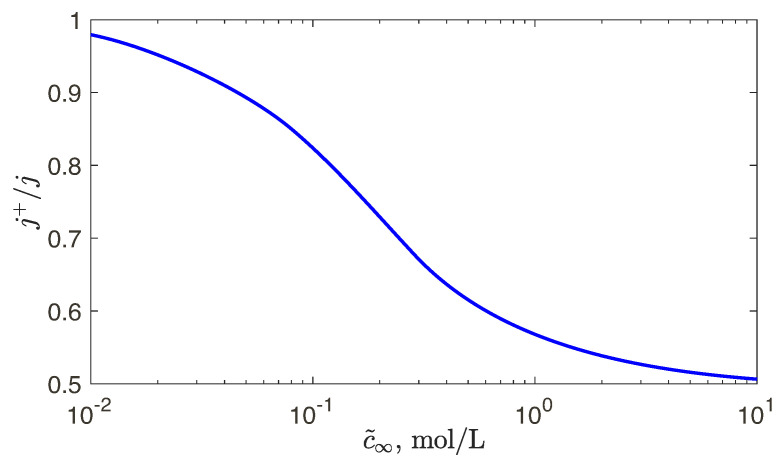
(Color online) Selectivity $j^{+}/\left(j^{+}-j^{-}\right)$ with the typical fixed charge density $\tilde{N}=0.8$ mol/L for the Nafion 120 membrane vs. the bulk concentration ${\tilde{c}}_{\infty }$.

We begin our analysis with an important quantity, the selectivity of the membrane, $j^{+}/j=j^{+}/\left(j^{+}-j^{-}\right)$, which shows deviation from the perfect membrane with $j^{+}/j=1$ and $j^{-}/j=0$. It was found that selectivity depends weakly on the voltage, but its dependence on the dimensionless fixed charge, $N=\tilde{N}/{\tilde{c}}_{\infty }$, is strong. For different standard membranes used in industry the dimensional fixed charge is always about 1 mol/L. Hence, it is instructive to plot the selectivity versus the bulk concentration ${\tilde{c}}_{\infty }$ instead of *N*. Such dependence for the Nafion 120 membrane is shown in Figure 3. For low-concentration salt solutions, ${\tilde{c}}_{\infty }<10^{-2}$ mol/L, the membrane with a good accuracy can be assumed perfect. Increasing the bulk salt concentration decreases the selectivity significantly; for ${\tilde{c}}_{\infty }=10$ mol/L it is only 0.5! Note that our numerical model allows to avoid painstaking experiments and, instead, seek selectivity theoretically.

**Figure 4:**
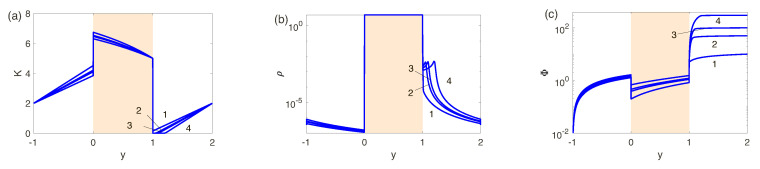
(Color online) Distributions of (**a**) the salt concentration $K=c^{+}-c^{-}$, (**b**) the charge density $\rho =c^{+}-c^{-}$, and (**c**) the electric potential Φ with respect to normal to the electrolyte-membrane interfaces for N=5 and 1: ΔV=10, 2: ΔV=50, 3: ΔV=100, and 4: ΔV=300.

Consider detailed results for three different membranes with the fixed dimensionless charge density *N*, N=5, N=0.5 and N=0.1 thus having characteristic ion-selectivities which are, respectively, close to perfect, intermediate and imperfect. In Figure 4a–c the dependence of the salt concentration *K*, the charge density ρ and the potential Φ on the spatial coordinate *y* for N=5 (perfect membranes or low-concentration electrolyte solutions)and four values of the voltage ΔV are presented. Pay attention to the jumps in salt concentration, charge density, and potential when crossing the electrolyte-membrane interface for all ΔV. This is characteristic of the Donnan enrichment/exclusion of membranes approaching perfectly ion-selective behavior. For the first values of ΔV, 10 and 50, corresponds to the underlimiting current regime, where ρ and *K* vary with distance practically linearly, excluding narrow O(ν) EDLs near phase interfaces. For the limiting current regime, ΔV=100 and 300, neither the behavior in the salt enriched region nor the porous membrane changes qualitatively. However, in the salt depleted region, the behavior of all the unknowns changes dramatically. For *K* a zone of practically zero salt concentration appears near y=1, which expands as the potential difference ΔV increases. In the ρ(y)-profile, a maximum appears in the space charge which increases and departs from the membrane with increasing ΔV. The potential distribution, Φ(y), along the membrane, shown in Figure 4c, has its own distinctive features. The potential drop inside the nanoporous membrane is almost linear, i.e., obeying Ohm’s law. The potential change in the enriched solution region is relatively small due to its good electrical conductivity. In the case of limiting current regimes, a jump in potential both in the membrane and in the zone of an enriched solution can be neglected in comparison with a change of the potential in the zone of a depleted solution. This is due to the fact that in the last zone the electric conductivity tends to zero at K→0.

**Figure 5:**
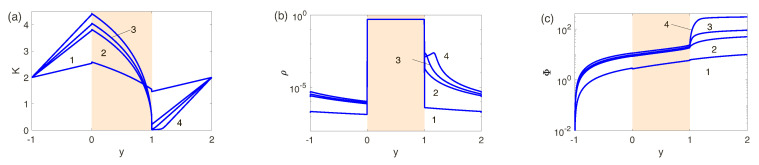
(Color online) Distributions of (**a**) the salt concentration $K=c^{+}-c^{-}$, (**b**) the charge density $\rho =c^{+}-c^{-}$, and (**c**) the electric potential Φ with respect to normal to the electrolyte-membrane interfaces for N=0.5 and 1: ΔV=10, 2: ΔV=50, 3: ΔV=100, and 4: ΔV=300.

In Figure 5a–c K(y), ρ(y) and Φ(y) for the intermediate value for the fixed charge, N=0.5, and four values of the voltage ΔV are shown. Qualitatively, the behavior is similar outside the membrane. However, the exclusion/enrichment effect inside the membrane decreases with decreasing *N*. Still, there is a small jump of the salt concentration *K* in the EDL of the enriched electrolyte solution. As a consequence of the reduced selectivity, at −1<y<1; the maximum of the charge in the ρ-distribution of the depleted region, 1<y<2, does not appear until higher voltages and becomes less profound. The zone of depleted solution in 1<y<2 becomes smaller and Φ(y)-distribution becomes smoother; specifically the transition between the membrane and the electrolyte near the interface y=0 does not have a jump. The steep gradient at y=1 remains, however, reduced in comparison to the perfect case due to the increased conductivity in the depleted region.

**Figure 6:**
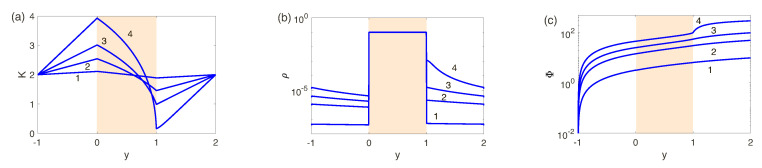
(Color online) Distributions of (**a**) the salt concentration $K=c^{+}-c^{-}$, (**b**) the charge density $\rho =c^{+}-c^{-}$, and (**c**) the electric potential Φ with respect to normal to the electrolyte-membrane interfaces for N=0.1 and 1: ΔV=10, 2: ΔV=50, 3: ΔV=100, and 4: ΔV=300.

In Figure 6a–c a smaller value of the fixed charge, N=0.1 (high-concentration electrolyte solutions), is considered. The most astonishing difference between large and small N (or low-concentration and high-concentration solutions) lies in absence of the salt depleted region. In turn, the maximum of the space charge, typical for low-concentration solutions, disappears (compare Figure 4a,b and Figure 6a,b). As a result, the gradient of Φ on the coordinate *y* in the depleted region decreases even further, see Figure 6c. Summarizing, we can say that for the small *N* (high-concentration electrolyte solutions) the difference between the underlimiting and limiting currents vanishes.

**Figure 7:**
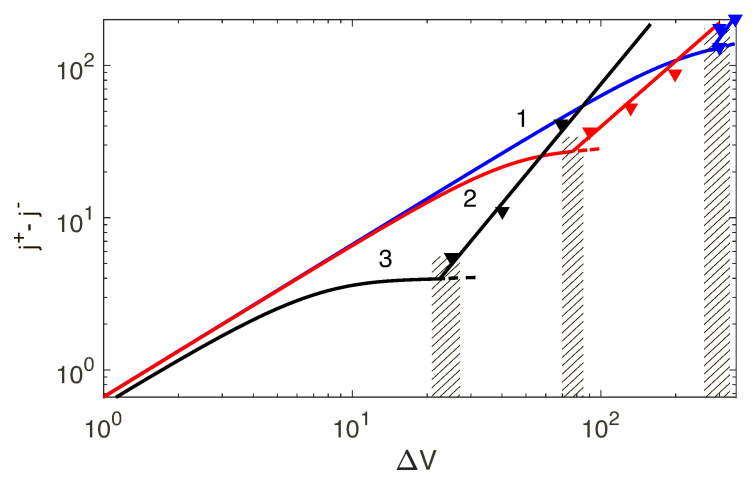
(Color online) VC characteristics. $j^{+}-j^{-}$ for 1: N=0.1, 2: N=0.5, 3: N=5. Shaded region corresponds to the range of critical $\Delta V^{*}$ for Da from 0 to $5\times 10^{-2}$.

Let us consider this fact from the view point of the voltage-current characteristic; the VC-dependence for the different N=0.1, 0.5 and 5 is shown in Figure 7. We point to a rather long segment of the limiting currents for N=5 (low-concentration electrolyte solutions) and its practical absence for N=0.1; there is no difference between the underlimiting ang limiting regimes for the high-concentration electrolytes. This fact was theoretically pointed out in the work by Rubinstain and Zaltzman [22]. We will return to this VC-characteristic in the next chapters.

### 3.2. Transition to the Overlimiting Currents

As was first discovered theoretically [11,12], the main reason for the transition to the overlimiting regimes is a special kind of electrohydrodynanic instability, the electrokinetic instability. In this subchapter, the transition to the overlimiting currents is considered from the viewpoint of classical linear stability theory. Assume that at some ΔV the 1D quiescent steady-state solution, described by Equations (34) and (35), is disturbed by small sinusoidal perturbations,

$$c^{\pm }=c_{0}^{\pm }+{\hat{c}}^{\pm }\exp\left(ikx+\lambda t\right),\;\Phi =\Phi_{0}+\hat{\Phi }\;exp\left(ikx+\lambda t\right),$$

$$U=\hat{U}\;exp\left(ikx+\lambda t\right),\;V=\hat{V}\;exp\left(ikx+\lambda t\right),$$

with the wave number *k* and the growth (decay) factor λ. These perturbations trigger a hydrodynamic flow so that now the velocity components $\hat{U}$ and $\hat{V}$ are nonzero. The subscript 0 is related to the mean 1D solution, the ‘^’, to perturbations. Upon substitution into the system (21)–(33), then linearizing with respect to perturbations and omitting the subscript 0 in the mean solution, we get the following system,

y=2:

(37) $$\hat{\Phi }=0,\;{\hat{c}}^{+}={\hat{c}}^{-}=0,\;\hat{V}=0,\;\frac{d\hat{U}}{dy}+ik\hat{V}=0,$$

2<y<1:

(38) $$\lambda {\hat{c}}^{+}+\hat{V}\frac{dc^{+}}{dy}=\frac{d}{dy}\left(c^{+}\frac{d\hat{\Phi }}{dy}+\frac{d\Phi }{dy}{\hat{c}}^{+}+\frac{d{\hat{c}}^{+}}{dy}\right)-k^{2}c^{+}\hat{\Phi }-k^{2}{\hat{c}}^{+},$$

(39) $$\lambda {\hat{c}}^{-}+\hat{V}\frac{dc^{-}}{dy}=\frac{d}{dy}\left(-c^{-}\frac{d\hat{\Phi }}{dy}-\frac{d\Phi }{dy}{\hat{c}}^{-}+\frac{d{\hat{c}}^{-}}{dy}\right)-k^{2}c^{-}\hat{\Phi }-k^{2}{\hat{c}}^{-},$$

(40) $$\nu^{2}\left(\frac{d^{2}\hat{\Phi }}{dy^{2}}-k^{2}\hat{\Phi }\right)={\hat{c}}^{-}-{\hat{c}}^{+},$$

(41) $$-ik\hat{\Pi }+\frac{d^{2}\hat{U}}{dy^{2}}-k^{2}\hat{U}=\frac{\kappa }{\nu^{2}}\left(c^{+}-c^{-}\right)ik\hat{\Phi },$$

(42) $$\frac{d\hat{\Pi }}{dy}+\frac{d^{2}\hat{V}}{dy^{2}}-k^{2}\hat{V}=\frac{\kappa }{\nu^{2}}\left({\hat{c}}^{+}-{\hat{c}}^{-}\right)\frac{d\Phi }{dy}+\frac{\kappa }{\nu^{2}}\left(c^{+}-c^{-}\right)\frac{d\hat{\Phi }}{dy},$$

$$\frac{d\hat{V}}{dy}+ik\hat{U}=0,$$

y=1:

(43) $$\hat{\Phi }{|}_{y=1+0}=\hat{\Phi }{|}_{y=1-0},\;\frac{d\hat{\Phi }}{dy}{|}_{y=1+0}=\frac{d\hat{\Phi }}{dy}{|}_{y=1-0},$$

(44) $${\hat{c}}^{\pm }{|}_{y=1+0}={\hat{c}}^{\pm }{|}_{y=1-0},\;\frac{d{\hat{c}}^{\pm }}{dy}{|}_{y=1+0}=\frac{d{\hat{c}}^{\pm }}{dy}{|}_{y=1-0},$$

$$\hat{U}{|}_{y=1+0}=\hat{U}{|}_{y=1-0},\;\hat{V}{|}_{y=1+0}=\hat{V}{|}_{y=1-0},\;-\hat{\Pi }{|}_{y=1+0}+\frac{d\hat{V}}{dy}{|}_{y=1+0}=-\hat{\Pi }{|}_{y=1-0}+\frac{d\hat{V}}{dy}{|}_{y=1-0},$$

(45) $$\frac{d\hat{U}}{dy}{|}_{y=1+0}+ik\hat{V}{|}_{y=1+0}=\frac{d\hat{U}}{dy}{|}_{y=1-0}+ik\hat{V}{|}_{y=1-0}-\frac{\beta }{Da}\hat{U}{|}_{y=1-0},$$

1<y<0:

(46) $$\lambda {\hat{c}}^{+}+\hat{V}\frac{dc^{+}}{dy}=\frac{d}{dy}\left(c^{+}\frac{d\hat{\Phi }}{dy}+\frac{d\Phi }{dy}{\hat{c}}^{+}+\frac{d{\hat{c}}^{+}}{dy}\right)-k^{2}c^{+}\hat{\Phi }-k^{2}{\hat{c}}^{+},$$

(47) $$\lambda {\hat{c}}^{-}+\hat{V}\frac{dc^{-}}{dy}=\frac{d}{dy}\left(-c^{-}\frac{d\hat{\Phi }}{dy}-\frac{d\Phi }{dy}{\hat{c}}^{-}+\frac{d{\hat{c}}^{-}}{dy}\right)-k^{2}c^{-}\hat{\Phi }-k^{2}{\hat{c}}^{-},$$

(48) $$\nu^{2}\left(\frac{d^{2}\hat{\Phi }}{dy^{2}}-k^{2}\hat{\Phi }\right)={\hat{c}}^{-}-{\hat{c}}^{+},$$

(49) $$-ik\hat{\Pi }+\frac{1}{Da^{2}}\hat{U}+\frac{d^{2}\hat{U}}{dy^{2}}-k^{2}\hat{U}=\frac{\kappa }{\nu^{2}}\left(c^{+}-c^{-}\right)ik\hat{\Phi },$$

(50) $$\frac{d\hat{\Pi }}{dy}+\frac{1}{Da^{2}}\hat{V}+\frac{d^{2}\hat{V}}{dy^{2}}-k^{2}\hat{V}=\frac{\kappa }{\nu^{2}}\left({\hat{c}}^{+}-{\hat{c}}^{-}\right)\frac{d\Phi }{dy}+\frac{\kappa }{\nu^{2}}\left(c^{+}-c^{-}\right)\frac{d\hat{\Phi }}{dy},$$

$$\frac{d\hat{V}}{dy}+ik\hat{U}=0,$$

y=0:

(51) $$\hat{\Phi }{|}_{y=-0}=\hat{\Phi }{|}_{y=+0},\;\frac{d\hat{\Phi }}{dy}{|}_{y=-0}=\frac{d\hat{\Phi }}{dy}{|}_{y=+0},$$

(52) $${\hat{c}}^{\pm }{|}_{y=-0}={\hat{c}}^{\pm }{|}_{y=+0},\;\frac{d{\hat{c}}^{\pm }}{dy}{|}_{y=-0}=\frac{d{\hat{c}}^{\pm }}{dy}{|}_{y=+0},$$

$$\hat{U}{|}_{y=+0}=\hat{U}{|}_{y=-0},\;\hat{V}{|}_{y=+0}=\hat{V}{|}_{y=-0},\;-\hat{\Pi }{|}_{y=+0}+\frac{d\hat{V}}{dy}{|}_{y=+0}=-\hat{\Pi }{|}_{y=-0}+\frac{d\hat{V}}{dy}{|}_{y=-0},$$

(53) $$\frac{d\hat{U}}{dy}{|}_{y=+0}+ik\hat{V}{|}_{y=+0}=\frac{d\hat{U}}{dy}{|}_{y=-0}+ik\hat{V}{|}_{y=-0}-\frac{\beta }{Da}\hat{U}{|}_{y=-0},$$

−1<y<0:

(54) $$\lambda {\hat{c}}^{+}+\hat{V}\frac{dc^{+}}{dy}=\frac{d}{dy}\left(c^{+}\frac{d\hat{\Phi }}{dy}+\frac{d\Phi }{dy}{\hat{c}}^{+}+\frac{d{\hat{c}}^{+}}{dy}\right)-k^{2}c^{+}\hat{\Phi }-k^{2}{\hat{c}}^{+},$$

(55) $$\lambda {\hat{c}}^{-}+\hat{V}\frac{dc^{-}}{dy}=\frac{d}{dy}\left(-c^{-}\frac{d\hat{\Phi }}{dy}-\frac{d\Phi }{dy}{\hat{c}}^{-}+\frac{d{\hat{c}}^{-}}{dy}\right)-k^{2}c^{-}\hat{\Phi }-k^{2}{\hat{c}}^{-},$$

(56) $$\nu^{2}\left(\frac{d^{2}\hat{\Phi }}{dy^{2}}-k^{2}\hat{\Phi }\right)={\hat{c}}^{-}-{\hat{c}}^{+},$$

(57) $$ik\hat{\Pi }+\frac{d^{2}\hat{U}}{dy^{2}}-k^{2}\hat{U}=\frac{\kappa }{\nu^{2}}\left(c^{+}-c^{-}\right)ik\hat{\Phi },$$

(58) $$\frac{d\hat{\Pi }}{dy}+\frac{d^{2}\hat{V}}{dy^{2}}-k^{2}\hat{V}=\frac{\kappa }{\nu^{2}}\left({\hat{c}}^{+}-{\hat{c}}^{-}\right)\frac{d\Phi }{dy}+\frac{\kappa }{\nu^{2}}\left(c^{+}-c^{-}\right)\frac{d\hat{\Phi }}{dy},$$

(59) $$\frac{d\hat{V}}{dy}+ik\hat{U}=0,$$

y=−1:

(60) $$\hat{\Phi }=0,\;{\hat{c}}^{+}={\hat{c}}^{-}=0,\;\hat{V}=0,\;\frac{d\hat{U}}{dy}+ik\hat{V}=0,$$

which is an eigenvalue problem for λ for a system of linear ordinary differential equations (ODEs). The coefficients of these equations depend on the solution of 1D problem (34) and (35). If real part of λ for all wave numbers *k* is negative, the 1D solution is stable; if real part of λ is positive for at least one *k*, the 1D solution is unstable and it gives the threshold of transition to the overlimiting currents.

The system has two small parameters before the highest order derivatives, the Debye number, ν, in the Poisson equation and the Darcy number, Da, in the Darcy–Brinkman equations. That is the very reason of creation of the thin boundary layers near the interfaces y=0 and y=1. In view of the foregoing, in each of the three layers—the region of the depleted solution, the membrane, and the region of the enriched solution, discretization was performed by the Galerkin method, and the Chebyshev polynomials, $T_{i}\left(z\right)$, were used as basis functions,

(61) $${\hat{c}}^{\pm }=\sum_{i}c_{i}^{\pm }T_{i}\left(z\right),\;\hat{\Phi }=\sum_{i}\Phi_{i}T_{i}\left(z\right),\;\hat{U}=\sum_{i}U_{i}T_{i}\left(z\right),\;\hat{V}=\sum_{i}V_{i}T_{i}\left(z\right)$$

The length of each layer is 1. Since the Chebyshev polynomials are defined in the interval −1<z<+1, the length of all three layers was doubled. The resolution of these polynomials increases as we approach the boundaries of the region; therefore, the Chebyshev polynomials ideally take into account the specifics of the problem. Individually, the Chebyshev polynomials do not satisfy the boundary conditions at the cathode and anode and the continuity conditions. Therefore, a τ-version of the Galerkin method was used.

The essence of the τ-version is that the relations (61) were substituted into the Equations (38)–(42), (46)–(53) and (54)–(59), and the condition of orthogonality was applied of the residual of the right-hand side of the equations. The last 22 conditions of orthogonality were excluded from the system and replaced by boundary conditions (37), (43)–(45), (51)–(52) and (60), where the Galerkin polynomials (61) were also substituted. Eventually, the eigenvalue problem for the ODE system was replaced by a generalized algebraic eigenvalue problem of matrices, as it follows,

det||λB+A||=0.

This problem was solved numerically by a standard QR-algorithm.The maximum dimension of the matrix reached 6000; we usually confined them to 2000.

The transition to the overlimiting currents is described by the following parameters: the voltage, ΔV, the fixed charged density, *N*, the Darcy number, Da, and the Brinkman coefficient, β (we recall, that the Debye number and the coupling coefficient were fixed, $\nu =5\times 10^{-4}$ and κ=0.1). The Brinkman coefficient β is determined by the texture properties of the nanoporous membranes and the size of its pores; this parameter varies from −1 to 1. Kuznetsov [35] showed that the influence of this parameter is insignificant. However, in his formulation the electrodynamical effects are not involved. Following [35], we also varied the parameter β in the above range, and found that the difference between solutions with β=−1, 0 and +1 is less than 1% for all the studied modes. Physically, this effect can be explained as follows: (a) For large values of *N*, the membrane is close to a perfect and, hence, the instability is dominated by the surface galvanosmotic slip velocity and must be independent of the membrane inner properties (see Ref. [22,23,25]) and, in particular, of β. (b) In the case of small *N*, the influence of the inner properties of the membrane as a whole becomes important. However, since for small *N* the electroconvection is largely caused by the volumetric residual charge in the electrolyte layer rather than the electroosmotic slip velocity (see [25]), again, influence of the inner properties of the membrane layer remains negligible. Hypothetically, the parameter β may be important for the equilibrium electrokinetic instability caused by the electroosmotic velocity, but such a regime was not found in the acceptable ranges of the parameters. Taking into account all this arguments, the results with β=0 are presented below.

**Figure 8:**
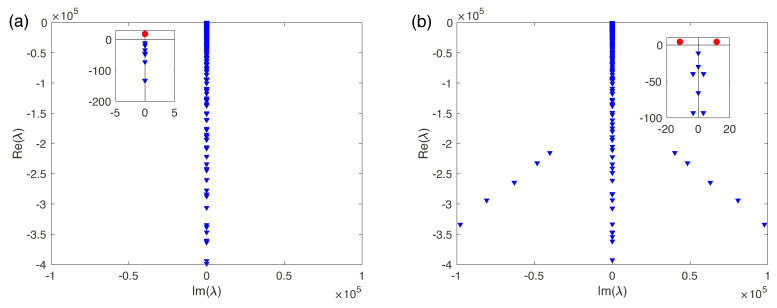
(Color online) Spectrum for the monotonic and oscillatory regimes for $Da=5\times 10^{-2}$: (**a**) N=5, ΔV=25, k=3 and (**b**) N=0.5, ΔV=90, k=2. Red circles stand for the unstable modes.

The discrete spectrum of eigenvalues $\lambda_{k}$ consists of both real and complex-conjugate eigenvalues. We will enumerate the eigenvalues according to their real part, $Re\{\lambda_{1}\}$$>Re\{\lambda_{2}\}$$>Re\{\lambda_{3}\}>\ldots$$>Re\{\lambda_{n}\}>\ldots$. When the first real eigenvalue $\lambda_{1}$ or the first pair of the complex-conjugate eigenvalues $Re\{\lambda_{1,2}\}$ crosses the imaginary axis of the complex λ-plane, the 1D steady-state solution looses its stability and induces transition to the overlimiting currents. For large *N*, the membrane approaches the perfect case and the spectrum is real (see Ref. [17]). In this case, the instability is monotonic. However, small *N* corresponds to the complex-conjugate pairs of eigenvalues and, hence, instability is oscillatory (Figure 8a,b, respectively). So, transition to the overlimiting regimes is realized through one of two scenarios: Figure 8a the monotonic transition for a perfect membrane (low-concentration electrolyte solutions), or Figure 8b the oscillatory one for the imperfect membranes (high-concentration electrolyte solutions).

**Figure 9:**
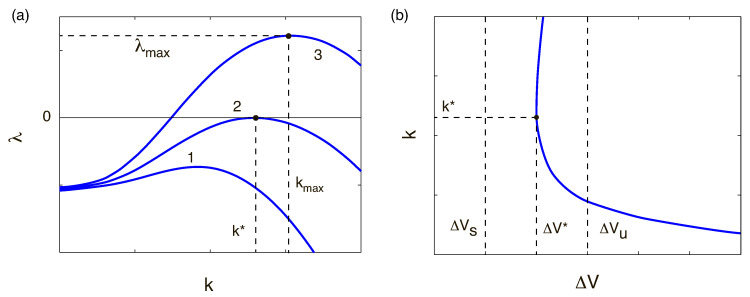
(Color online) Schematical dependencies. (**a**) The growth (decay) rate λ vs. the wave number *k* for three values of the parameter *N*, line 1 corresponds to $\Delta V=\Delta V_{s}<\Delta V^{*}$ on figure (**b**), line 2: $\Delta V^{*}$, line 3: $\Delta V=\Delta V_{u}>\Delta V^{*}$. (**b**) Marginal stability curves in the plain ΔV−k. $\Delta V_{s}$ corresponds to stable ΔV, $\Delta V^{*}$: critical ΔV and $\Delta V_{u}$: unstable ΔV.

The instability and transition are connected with the largest eigenvalue, $\lambda =\lambda_{1}$. For stable $\Delta V=\Delta V_{s}<\Delta V^{*}$λ(k) decays for all wave numbers; for the unstable $\Delta V=\Delta V_{u}>\Delta V^{*}$ there is a window of unstable wave numbers with λ>0; the for critical case $\Delta V=\Delta V^{*}$ there is critical $k=k^{*}$ with $\lambda (k^{*})=0$, while all other wave numbers are stable, λ(k)<0 for $k\neq k^{*}$. The critical value $\Delta V^{*}$ gives the electrokinetic instability threshold and critical wave number $k^{*}$ and the boundary between the one-dimensional underlimiting or limiting regimes and two-dimensional microvortex regime, see Figure 9a,b.

Let us return to the VC-characteristics for the underlimiting and limiting currents from the previous chapter (Figure 7), described by the 1D steady-state quiescent equilibria. The instability violates this state of equilibrium and leads to the ovelimiting regimes. While the one-dimensional equilibria do not depend on the hydromechanics of the process and thus on the Darcy number, the transition point $\Delta V^{*}$ does in fact depend on it (see shaded areas in the Figure). The portion of the VC-characteristic for the supercritical voltage, $\Delta V>\Delta V^{*}$, can be described only by the direct numerical simulation of the problem in the full nonlinear formulation (21)–(33). $\Delta V^{*}$ is a function of the fixed charge *N*: (a) For sufficiently large *N*, *N* from 5 to 10, the membrane becomes practically perfect (low-concentration electrolyte solutions) and the critical voltage ceases to depend on *N*. (b) For a small *N* (high-concentration electrolyte solutions) $\Delta V^{*}$ increases with decreasing of *N*. An interesting feature of high-concentration electrolyte solutions is that the flat portion of the VC-characteristic, responsible for the limiting currents, disappears and transition to the overlimiting regimes happens right from the underlimiting regimes, thus bypassing the portion of the current saturation in the VC curve. This fact was first elucidated in the works [22,23] for a simplified solution for small *N*.

**Figure 10:**
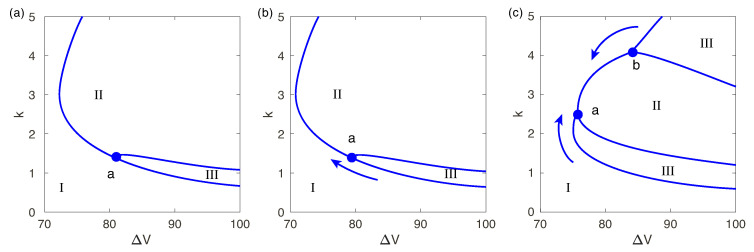
(Color online) Marginal stability curves at N=0.5 and for different Darcy numbers Da: (**a**): $Da=10^{-3}$, (**b**): $Da=5\times 10^{-3}$, (**c**): $Da=5\times 10^{-2}$. Region I corresponds to stable eigenvalues, there is one unstable eigenvalue in region II and two unstable eigenvalues in region III.

Marginal stability curves depend on the parameters *N* and Da in a rather sophisticated way. In Figure 10a a typical marginal stability curve is shown for a small but nonzero Da. For a small subcriticality there is only one unstable mode, region II. However, with increasing voltage a thin region III with a second unstable mode appears near the lower branch of the marginal curve (the long-wave mode). The left boundary of this region is determined by the point “a”. With increasing Da-number, this point moves towards the nose of the stability curve. At the same time a point “b” on the upper branch of the stability curve appears; it characterizes an additional unstable region III which arises for the large wave numbers (the short-wave mode). As the Darcy number increases, both points move towards each other and merge at a critical voltage $\Delta V^{*}$, see Figure 10b,c. With increasing of *N* region III disappears, and the instability is governed by only one unstable real eigenvalue.

**Figure 11:**
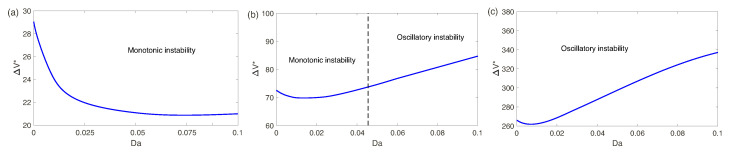
(Color online) The critical voltage, $\Delta V^{*}$, versus the Darcy number Da. (**a**) N=5, (**b**) N=0.5, and (**c**) N=0.1. The dashed vertical line separates regions of the monotonic and oscillatory instabilities.

Dependence of the critical voltage $\Delta V^{*}$ on both *N* and Da is shown in Figure 11. The dependence of $\Delta V^{*}$ on Da is not monotonic; it has a minimum: at N=5 at $Da=5.1\times 10^{-2}$, at N=0.5 at $Da=10^{-2}$ and at N=5 at $Da=8.1\times 10^{-3}$. Existence of this minimum is connected with the fact of interplay of the short-wave and long-wave modes.

**Figure 12:**
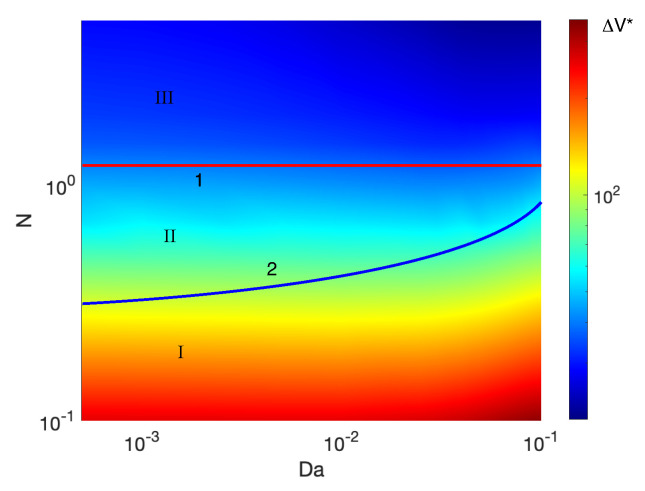
(Color online) Map of regimes. Curve 1 separates the region of the equilibrium (II) and nonequilibrium (III) instabilities. This curve is determined by the 1D solution and, so, does not depend on the Darcy number Da. Curve 2 separates the regions of oscillatory (I) and monotonic (II, III) instabilities. The critical value ΔV is highlighted by the background color.

The results are summarized on the mode map, Figure 12, where a composite dependence of $\Delta V^{*}$ on *N* and Da is shown. The critical voltage is highlighted by the background color, see to the left of the map. The nonequilibrium instability is characteristic for the large *N* (perfect membranes and low-concentration electrolyte solutions). The equilibrium instability begins to prevail with decreasing of *N* (imperfect membranes and high-concentration electrolyte solutions). The critical voltage, $\Delta V^{*}$, is increasing with decreasing fixed charge *N*. An increase in the Darcy number enhances the growth of critical voltage $\Delta V^{*}$. Instability is monotonic for perfect membranes and this fact is independent of the Darcy number. As the Darcy number increases, the transition region in *N* between monotonic and oscillatory instabilities narrows.

### 3.3. Direct Numerical Sumulation of Well-Developed Overlimiting Regimes

In order to find numerical solutions of the full nonlinear system (21)–(33) for the developed overlimiting currents, the quasi-spectral discretization in space was used. With respect to the independent variable *x* directed along the membrane, the unknown concentrations of anions and cations, the electric potential, and the velocity components in all three layers were represented as Fourier series:

(62) $$F\left(t,x,y\right)=\sum_{n=0}^{M_{1}}\;F_{n}\left(t,y\right)\;exp\left(inkx\right),$$

where *k* is a basic wave number for a characteristic length of the channel, 2π/k and nk, n=2,3,… are the overtones. Therefore, for each layer, $M_{1}$ one-dimensional unsteady problems for the each harmonic, $n=0,1,\ldots M_{1}-1$ arise,

$$\frac{\partial c_{n}^{\pm }}{\partial t}+i\frac{nk}{2}U_{n/2}c_{n/2}^{\pm }+V_{n/2}\frac{\partial c_{n/2}^{\pm }}{\partial y}=$$

(63) $$\pm \frac{\partial }{\partial y}\left(c_{n/2}^{\pm }\frac{\partial \Phi_{n/2}}{\partial y}\right)\pm \frac{n^{2}k^{2}}{2}c_{n/2}^{\pm }\Phi_{n/2}+\frac{\partial^{2}c_{n}^{\pm }}{\partial y^{2}}-n^{2}k^{2}c_{n}^{\pm },$$

(64) $$\nu^{2}\left[\frac{\partial^{2}\Phi_{n}}{\partial y^{2}}-n^{2}k^{2}\Phi_{n}\right]=c_{n}^{-}-c_{n}^{+},$$

(65) $$ink\Pi_{n}=\frac{\partial^{2}U_{n}}{\partial y^{2}}-n^{2}k^{2}U_{n}+ink\frac{\kappa }{\nu^{2}}\Phi_{n/2}\left(c_{n/2}^{-}-c_{n/2}^{+}\right),$$

(66) $$\frac{\partial \Pi_{n}}{\partial y}=\frac{\partial^{2}V_{n}}{\partial y^{2}}-n^{2}k^{2}V_{n}+\frac{\kappa }{\nu^{2}}\frac{\partial \Phi_{n/2}}{\partial y}\left(c_{n/2}^{-}-c_{n/2}^{+}\right),\;\frac{\partial U_{n}}{\partial y}+inkV_{n}=0.$$

The problem in the membrane layer is now formulated as,

$$\frac{\partial c_{n}^{\pm }}{\partial t}+i\frac{nk}{2}U_{n/2}c_{n/2}^{\pm }+V_{n/2}\frac{\partial c_{n/2}^{\pm }}{\partial y}=$$

(67) $$\pm \frac{\partial }{\partial y}\left(c_{n/2}^{\pm }\frac{\partial \Phi_{n/2}}{\partial y}\right)\pm \frac{n^{2}k^{2}}{2}c_{n/2}^{\pm }\Phi_{n/2}+\frac{\partial^{2}c_{n}^{\pm }}{\partial y^{2}}-n^{2}k^{2}c_{n}^{\pm },$$

(68) $$\nu^{2}\left[\frac{\partial^{2}\Phi_{n}}{\partial y^{2}}-n^{2}k^{2}\Phi_{n}\right]=c_{n}^{-}-c_{n}^{+},\;n\neq 0,$$

for zero harmonic, n=0,

(69) $$\nu^{2}\frac{\partial^{2}\Phi_{0}}{\partial y^{2}}=c_{0}^{-}-c_{0}^{+}+N,$$

and the Darcy–Brinkman equations turn into,

(70) $$ink\Pi_{n}=-\frac{1}{Da^{2}}\;U_{n}+\frac{\partial^{2}U_{n}}{\partial y^{2}}-n^{2}k^{2}U_{n}+ink\frac{\kappa }{\nu^{2}}\Phi_{n/2}\left(c_{n/2}^{-}-c_{n/2}^{+}\right),$$

(71) $$\frac{\partial \Pi_{n}}{\partial y}=-\frac{1}{Da^{2}}\;V_{n}+\frac{\partial^{2}V_{n}}{\partial y^{2}}-n^{2}k^{2}V_{n}+\frac{\kappa }{\nu^{2}}\frac{\partial \Phi_{n/2}}{\partial y}\left(c_{n/2}^{-}-c_{n/2}^{+}\right),\;\frac{\partial U_{n}}{\partial y}+inkV_{n}=0.$$

Then, for each function $F_{n}\left(t,y\right)$, discretization by *y* was carried out using the Galerkin method with the choice of Chebyshev’s polynomials $T_{n}\left(z\right)$ as basis functions. The *y*-coordinate was stretched twice to be mapped to −1<z<1,

(72) $$F_{n}=\sum_{j=0}^{M_{2}-1}F_{j,n}\left(t\right)T_{j}\left(z\right).$$

Chebyshev’s polynomials are well suited to our problem, since they have an accumulation of zeros and therefore a high resolution near the Debye and Darcy layers. Substituting the finite Chebyshev series into the system (63)–(71) and using the Lanczos τ method (see [36,37]) to satisfy the boundary conditions (24)–(30) leads to a system of coupled ODEs for the unknown Galerkin coefficients $c_{mn}^{\pm }\left(t\right)$ and two systems of linear algebraic equations with respect to $\Phi_{m,n}$ and $\Psi_{m,n}$ for the each layer. To obtain these systems, all nonlinear algebraic operations are executed in physical space, in the collocation points, while derivatives with respect to both spatial variables *x* and *y* are calculated in the space of the Galerkin coefficients. Derivatives of the Chebyshev polynomials are calculated by means of the collocation matrix method (see [36]). The connection between the collocation points and the Galerkin coefficients is performed by means of the fast Fourier transform.

A special method is developed to integrate the system in time. The numerical solution of the problem turned out to be very computationally costly. Therefore (a) the parallelization of the problem was applied on the Lomonosov supercomputer of the Moscow State University, (b) a special numerical scheme was used to discretize the problem in time. The second order Adams-Bashforth scheme for nonlinear terms along with the Crank-Nicholson scheme for linear terms were used in our work [13] for the perfect one-layer membrane. In order to significantly accelerate the calculations this method was changed. The system of ODE’s with respect to $dF_{j,n}/dt$ was integrated by the special third-order Runge-Kutta semi-implicit scheme adapted from the work [38].

“Room disturbances” as conditions at t=0 and initial stage of their evolution. At t=0 electroneutrality conditions were used, $c^{-}=c^{+}=1$ for the electrolyte layers and $c^{-}=c^{+}-N=1$ in the membrane layer. These electroneutral conditions always contain small random (thermal) perturbations at a minimum. Sometimes they are called “room disturbances”. To mimic the initial small-amplitude broad-banded white noise the initial conditions are presented in the form,

(73) $$t=0:\;c^{+}=1+\sum_{m=1}^{M}\;c_{\left(m\cdot k\right)}^{+}\;e^{imkx+\theta_{m}^{+}},\;c^{-}=1+\sum_{m=1}^{M}\;c_{\left(m\cdot k\right)}^{-}\;e^{imkx+\theta_{m}^{-}},$$

which is consistent with the decomposition (62). The amplitudes of these harmonics ${\hat{c}}^{\pm }$ are assumed to be small and their absolute value and phase $\theta_{m}^{\pm }$ for the each harmonic mk set by a random number generator uniformly distributed in the region (0,2π). The simulations were carried out for amplitude of the harmonic disturbance varying from $10^{-5}$ to $10^{-7}$, with most at $10^{-6}$. However, changing the amplitude did not change the results, but only the saturation time. Two basis wave numbers were taken; the rough calculations were done at k=1, but for more precise calculations k=0.5 were taken. In particular k=0.5 were taken for the evaluation of critical $\Delta V^{*}$ and $k^{*}$. Only the ion transport equations contain time derivatives and, hence, require the initial conditions.

**Figure 13:**
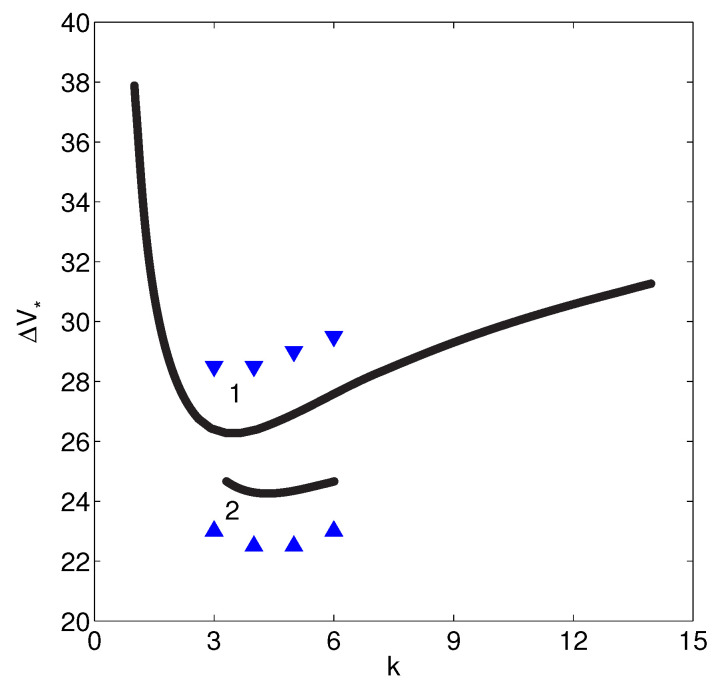
(Color online) Marginal stability curves according to the linear stability theory (the solid lines) and the DNS (the triangles), $Da=10^{-2}$. 1: N=10 and 2: N=20.

The initial evolution of the entire spectrum of small-amplitude harmonics is then described by the linear stability theory (see the previous Chapter). The amplitude of each harmonic mk, m=1,2,…, for the stable wavenumbers is exponentially decaying, while for the unstable wavenumbers it is growing. As it follows from the linear stability theory, evolution of each harmonic takes place independently from each other and is described by the eigenvalue problem (37)–(60). For the critical conditions $\Delta V=\Delta V^{*}$ there is a critical wave number $k^{*}=m^{*}\;k$, for which the amplitude does not change in time. Amplitudes of all other harmonics, $m\neq m^{*}$, are exponentially decaying in time. Comparison between the linear stability theory and our direct numerical calculation is presented in Figure 13 for two values of the fixed charge *N*. According the linear theory $\Delta V_{1}^{*}\approx 25.8$, $k_{1}^{*}\approx 3.42$ and $\Delta V_{2}^{*}\approx 24.2$, $k_{2}^{*}\approx 4.49$, while according to the DNS $\Delta V_{1}^{*}\approx 28.5$$k_{1}^{*}\approx 3.40$ and $\Delta V_{2}^{*}\approx 22.5$, $k_{2}^{*}\approx 4.50$. It shows reasonably good match between the linear stability theory and the direct numerical simulation of the full nonlinear system (21)–(33).

**Figure 14:**
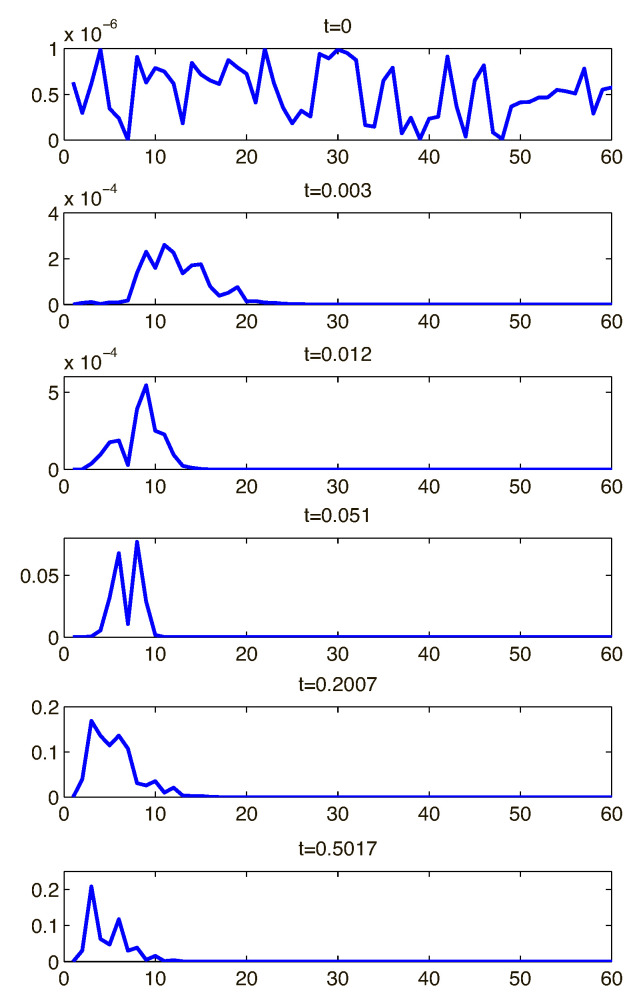
(Color online) Time evolution of the power spectrum amplitude versus wavenumber. ΔV=40, N=5 and $Da=10^{-3}$.

Typical evolution of the initial small-amplitude broad-banded white noise for a nonzero supercriticality $\Delta V-\Delta V^{*}$ is shown in Figure 14. k=0.5 was taken as a basic wave number, so that the harmonics, involved in the calculation, were $\left(0,k,2\;k,3\;k,\ldots ,M_{1}\;k\right)$ with $M_{1}=128$. Evolution begins with a random distribution of the amplitudes of these harmonics versus the wave number, t=0. At t=0.003 the linearly stable harmonics decayed. Amplitude of the linearly unstable harmonics, in the wave number window from 5 to 20, increased about 200 times. The amplitude of the “surviving” harmonics was still small and they continued to develop according to the linear stability theory, exponentially growing in time. At t=0.5017 the initial small-amplitude broad-banded white noise was filtered into practically monochromatic disturbances with a dominant wave number $k_{max}=3$, corresponded to the maximum growth rate $\lambda_{max}=\lambda \left(k_{max}\right)$ according to the linear theory.

Note, that the primary band focuses due to the filtering until the amplitudes of the disturbances are small. With amplitudes growing, the nonlinear effects began to corrupt this linear filtering. Overtone $3k_{\max}=6$ and and zero-frequency bands appeared, as seen in the Figure 14 at t=0.5017. The exponential growth downstream was also saturated by this nonlinear interaction.

### 3.4. The Nonlinear Stage of Evolution

The next stages of evolution are nonlinear processes with eventual saturation of the disturbance amplitude. For a small supercriticality $\Delta V-\Delta V_{*}$, the nonlinear saturation leads to steady periodic electroconvective vortices along the membrane surface. With increasing supercriticality the attractor can be described as a structure of periodically oscillating vortices. With a further increase in supercriticality, the behavior eventually becomes chaotic in time and space.

**Figure 15:**
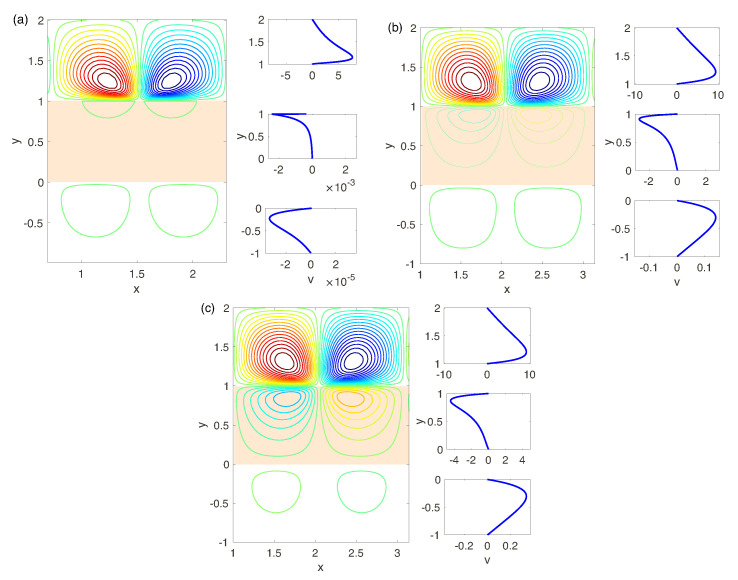
(Color online) (to the left) The stream function Ψ(x,y) and (to the right) the vertical component of velocity in the middle of the channel, ${V|}_{x=0}$. The parameters: N=5 and different Da and ΔV: (**a**): $Da=5\times 10^{-4}$ and ΔV=30, (**b**): $Da=5\times 10^{-3}$ and ΔV=25, (**c**): $Da=10^{-2}$ and ΔV=25.

**Figure 16:**
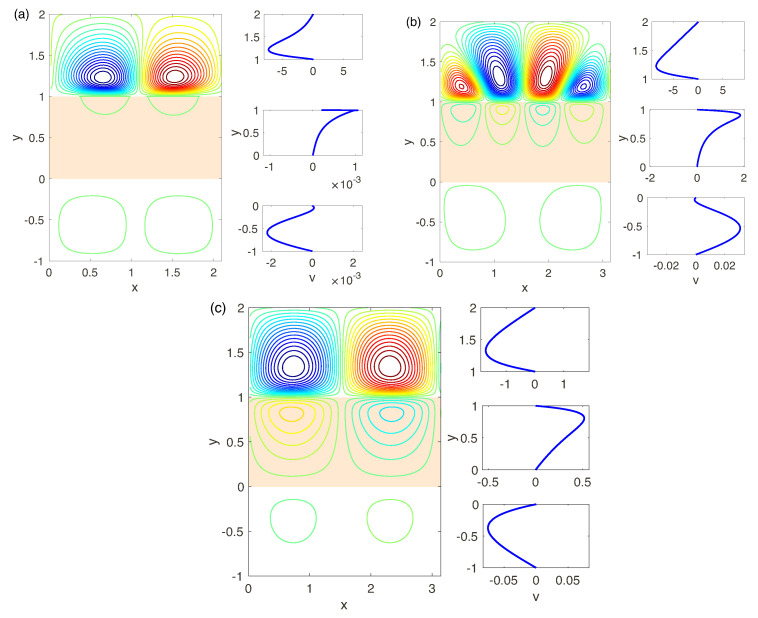
(Color online) See caption for Figure 15. The parameters: N=0.5, ΔV=90 and different Da : (**a**): $Da=5\times 10^{-4}$, (**b**): $Da=5\times 10^{-3}$, (**c**): $Da=10^{-2}$.

**Figure 17:**
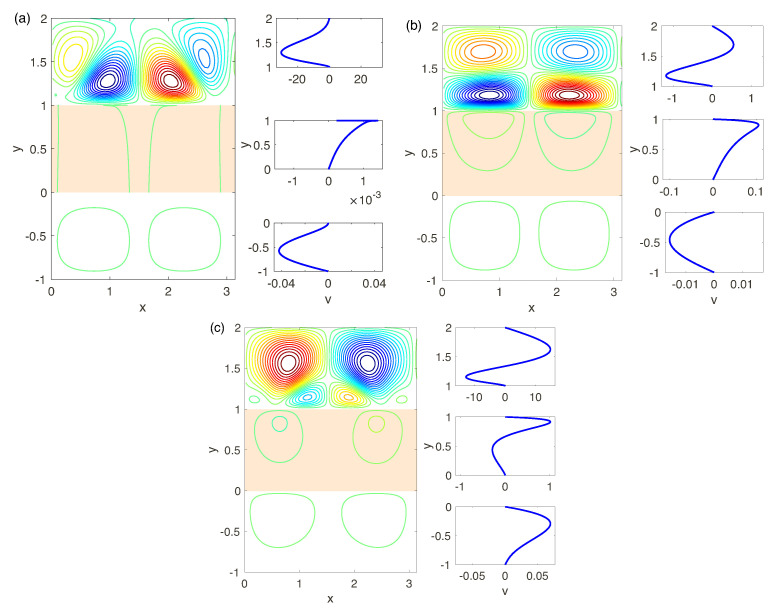
(Color online) See caption for Figure 15. The parameters: N=0.1 and different Da and and ΔV: (**a**): $Da=5\times 10^{-4}$ and and ΔV=300, (**b**): $Da=5\times 10^{-3}$ and ΔV=300, (**c**): $Da=10^{-2}$ and ΔV=350.

Established solutions are shown in Figure 15, Figure 16 and Figure 17, where snapshots of the stream lines Ψ(x,y) are presented. All the calculations are for small supercriticalities $\Delta V-\Delta V^{*}$, so that the attractor at t→∞ is regular.

We begin the discussion with the simplest case of the perfect membranes (low-concentration electrolyte solutions) for different Darcy numbers and voltage, see Figure 15a–c. According to the results of the linear stability analysis, the attractor corresponds to a pair of steady vorticies for all the Darcy numbers. For very small Da, Figure 15a, despite of strong instability and hydrodynamic movement in the depleted electrolyte layer, the membrane and enriched electrolyte layers practically do not react to it and velocity field in the regions 1<y<2 and 0<y<1 is rather weak. With the Darcy number increasing, see Figure 15b,c, the character of the vortex pair in the depleted region does not change much, but even small increasing of Da leads to a rise in the velocity field in the layers 0<y<1 and −1<y<0, comparable with the one in the depleted region. With increasing of the supercriticality $\Delta V-\Delta V^{*}$, the regime of regular microvortices, through several secondary instabilities and transitions, changes to a chaotic regime. These instabilities and transitions qualitatively are the same, as described in Ref. [17] for N=∞. Eventually note, that the charge field, $\rho =c^{+}-c^{-}$, near the electrolyte -membrane interface y=1 acquires a typical spike-like distribution with an angle at the apex of the spike about 120°, see Ref. [17], Figure 10 (the charge distribution is not presented in this work).

The results of the previous chapter on linear stability show the complex hydrodynamic behavior and, in particular, the appearance of several unstable modes and the change of the monotonic nature of the instability to the oscillatory one at decreasing of the fixed charge *N*, see Figure 11. The established regimes for N=0.5, for a small supercriticality $\Delta V-\Delta V^{*}$ and different Darcy numbers are presented in Figure 16. Again, for very small Da the picture is similar with N=5 and the attractor is a pair of steady vorticies, Figure 16a. With increasing of Da there is a possibility either of oscillatory or monotonic instabilities, see Figure 16b,c. The velocity field in the electrolyte depleted region initiates the hydrodynamic movement in the membrane and the electrolyte enriched region. The latter has the same order of magnitude as in the layer 1<y<2.

According to the linear analysis the stability for N=0.1 is always oscillatory. The nonlinear direct numerical simulation confirms this fact. Its resalts are presented in Figure 17. For all Da the instability is caused by a complex-conjugate pair and is thus oscillatory. Several vortices appear in the depleted region, 1<y<2, the complex behavior begins already for rather small supercriticality. With further very small increasing of ΔV the dynamics becomes chaotic.

For sufficiently large Da, the hydrodynamics in all three regions (electrolyte/membrane /electrolyte) begin to be linked and the analysis presented above, which affects only the depleted zone of the electrolyte and the membrane region, while ignoring the enriched zone, ceases to adequately reflect the behavior of the system.

## 4. Conclusions

Using a three layer composite electrolyte–nanoporous membrane–electrolyte model system, three characteristic regimes of VC response for imperfect and perfect ion selective membranes were studied numerically: the underlimiting, the limiting and the overlimiting. Mathematically, the Nernst–Planck–Poisson–Stokes system was taken in the electrolyte layers and the Darcy–Brinkman approach was employed in the membrane layer. To avoid numerical difficulties connected with the Debye and Darcy singularities, the quasi-spectral Galerkin method was applied. The transition to the overlimiting currents was assumed to be connected with the electrokinetic instability. The threshold of the transition was studied by the linear stability theory and the well-developed overlimiting currents were a subject of the DNS the full nonlinear system of equations. It was found that for a large fixed charge density of the membrane, it operates as a perfect membrane, while for a small fixed charge density the behavior of the imperfect membrane is much more sophisticated. In particular, the direct transition from the underlimiting regimes to the overlimiting ones, bypassing the limiting currents, was found to be possible for imperfect membranes. The transition to the overlimiting currents for the perfect membranes is monotonic, while for the imperfect ones it is oscillatory. Despite the fact that velocities in the porous membrane are much smaller than in the electrolyte region, they could dramatically influence to the transition to the overlimiting regimes. Increasing Darcy number generally speaking introduces more oscillatory instability into the system and consequently results in more complex hydrodynamics. Moreover, the results of the preceding analysis can be experimentally verified in a rather straightforward manner by observation of the nature of the instability in a micro-nanochannel system. Such a system has already been employed to investigate the effects of imperfect ion transport on the transient regime [39]. The Darcy number in such systems can be controlled to reasonably observe both oscillatory and monotonic regimes.
